# Supplementary material for: Association between Rumen Microbiota and Marbling Score in Korean Native Beef Cattle
Source: Animals (Basel). 2020 Apr 19;10(4):712. doi: 10.3390/ani10040712 (PMC7222830; doi:10.3390/ani10040712)
Supplement: Supplementary file 1 [file animals-10-00712-s001.pdf]

animals

MDPI

Article

Association between Rumen Microbiota and Marbling Score in Korean Native Beef Cattle

Minseok Kim [1,2,†], Tansol Park [3,†], Jin Young Jeong [1], Youlchang Baek [1] and Hyun-Jeong Lee [4,*]

1. Animal Nutrition & Physiology Team, National Institute of Animal Science, Wanju 55365, Republic of Korea; mkim2276@gmail.com (M.K.); jeong73@korea.kr (J.Y.J); chang4747@korea.kr (Y.B.)
2. Department of Animal Science, College of Agriculture and Life Sciences, Chonnam National University, Gwangju 61186, Republic of Korea; mkim2276@gmail.com
3. Department of Animal Sciences, The Ohio State University, Columbus, OH 43210, USA; tansol1719@gmail.com
4. Dairy Science Division, National Institute of Animal Science, Cheonan 31000, Republic of Korea; hyunj68@korea.kr

* Correspondence: hyunj68@korea.kr

† These authors contributed equally to this work.

Supplementary materials

Table S1. Feed and nutrient composition of diets during the early weaning period, growing and fattening stages.

Early weaning diet		
Item	Concentrate, %[1]	Starch 30%[2]
Corn	13.00	30.40
Wheat	10.00	10.00
Beet pulp pellet	5.00	5.00
Coconut oil meal	2.50	-
Gluten feed	6.00	3.00
Rice hull	1.00	1.00
Wheat bran	32.00	14.00
Wheat fl off	5.00	5.00
Almond shell	3.00	2.89
Soy hull	-	3.74
Rapeseed meal	2.00	2.00
Soybean meal	7.09	11.30
DDGS[3]	3.00	3.00
Limestone	3.00	1.97
Molasses cane	3.00	3.00
Salt	0.60	0.60
Mineral premix	0.20	0.20
Vitamin premix	0.12	0.12
Others	3.49	2.78

	Growing stage	Early fattening stage	Late fattening stage
	%	%	%
Gluten feed	4.83	12.46	13.49
Molasses cane	4.48	3.74	5.06
Wheat bran	42.44	24.92	16.86
Coconut kernel meal	3.12	7.48	6.74
Corn gluten meal	0	2.49	4.27
Corn flakes	31.25	39.00	45.52
Palm meal	10.70	7.48	5.62
Vitamin premix	0.45	0.12	0.11
Limestone	2.33	1.37	1.32
Salt	0.40	0.44	0.51
Sodium bicarbonate	0	0.50	0.50
Oat grass	33.95	3.02	0
Rice straw	0	16.72	11.03
**Nutrient composition (% of DM)**			
Crude ash	6.96	5.68	5.52
Crude protein	15.10	16.55	16.20
Ca	0.89	0.68	0.42
P	0.59	0.55	0.55
NDF	27.67	23.83	21.86

Nutrient composition (% of DM)		
Dry matter	87.88	87.49
Crude protein	15.00	15.00
Ether extract	2.81	2.71
Crude fiber	8.54	8.03
Crude ash	8.83	6.84
Ca	1.36	1.00
P	0.56	0.44
NFE	52.72	54.96
NFC	32.33	37.77
ADF	10.58	10.00
NDF	28.91	25.18
TDN	67.84	72.41

ADF	10.50	9.97	7.89
TDN	70.59	73.90	77.86

[1] Concentrate diet fed only to imprinting calves of T1, T2 and T3 dietary treatments from weaning period at 15±4 days of age to 85±4 d of age.

[2] Starch diet fed only to imprinting calves of T3 dietary treatment from weaning at 15±4 days of age to 85±4 d of age.

Control, mother milk + roughage; T1, milk replacement + concentrate; T2, milk replacement + concentrate + roughage (Timothy); T3, milk replacement + concentrate + 30% starch.

[3] Dried distillers grains with solubles.

Table S2. Pairwise PERMANOVA results of early weaning diet effect on the rumen microbiota of Hanwoo steers.

A. Based on the unweighted UniFrac distances

Group 1	Group 2	Permutations	pseudo-F	*Q*-value
Control	T1	999	0.8505	0.8120
	T2	999	1.1740	0.6405
	T3	999	1.4720	0.2040
T1	T2	999	0.8984	0.8120
	T3	999	1.0097	0.6405
T2	T3	999	1.4419	0.2040

B. Based on the weighted UniFrac distances

Group 1	Group 2	Permutations	pseudo-F	*Q*-value
Control	T1	999	1.2209	0.5100
	T2	999	1.3970	0.5055
	T3	999	2.6229	0.2160
T1	T2	999	0.7512	0.8940
	T3	999	1.1916	0.5055
T2	T3	999	1.3069	0.5055

Table S3. DADA2 denoising statistics of 16S rRNA gene amplicon sequencing variants from the rumen of Korean beef cattle resulting in different marbling score groups.

Cow ID	Marbling score	Marbling score groups	No. of input paired reads	Quality filtered reads	Denoised reads	Merged reads	Chimera-filtered reads	Taxa-filtered*
1000	3	LMS	242,981	147,841	140,264	102,560	86,945	86,672
3145	3	LMS	178,845	115,258	110,044	84,766	78,274	78,006
3159	2	LMS	220,505	138,029	134,313	114,078	87,803	85,265
4313	3	LMS	229,278	150,047	142,230	105,174	94,583	94,371
4326	3	LMS	186,729	119,469	113,382	87,618	80,005	79,490
1408	4	LMS	237,736	155,974	147,654	109,958	98,265	98,190
2696	4	LMS	206,219	131,346	124,836	95,125	83,645	83,517
1336	7	HMS	216,520	138,876	132,026	98,443	86,462	85,804
2276	7	HMS	216,763	141,260	133,857	99,943	90,111	90,053
2699	7	HMS	210,759	131,814	124,527	90,950	83,542	83,007
4316	7	HMS	245,960	159,378	151,103	115,089	100,960	100,187
3157	8	HMS	242,882	150,170	141,512	101,214	91,134	90,438
4424	8	HMS	227,655	147,679	140,381	104,621	92,366	92,210
4880	8	HMS	228,848	142,235	133,871	96,760	87,191	87,109

* Amplicon sequencing variants (ASVs) identified as “mitochondria,” “chloroplast,” and “Unassigned” were filtered out.

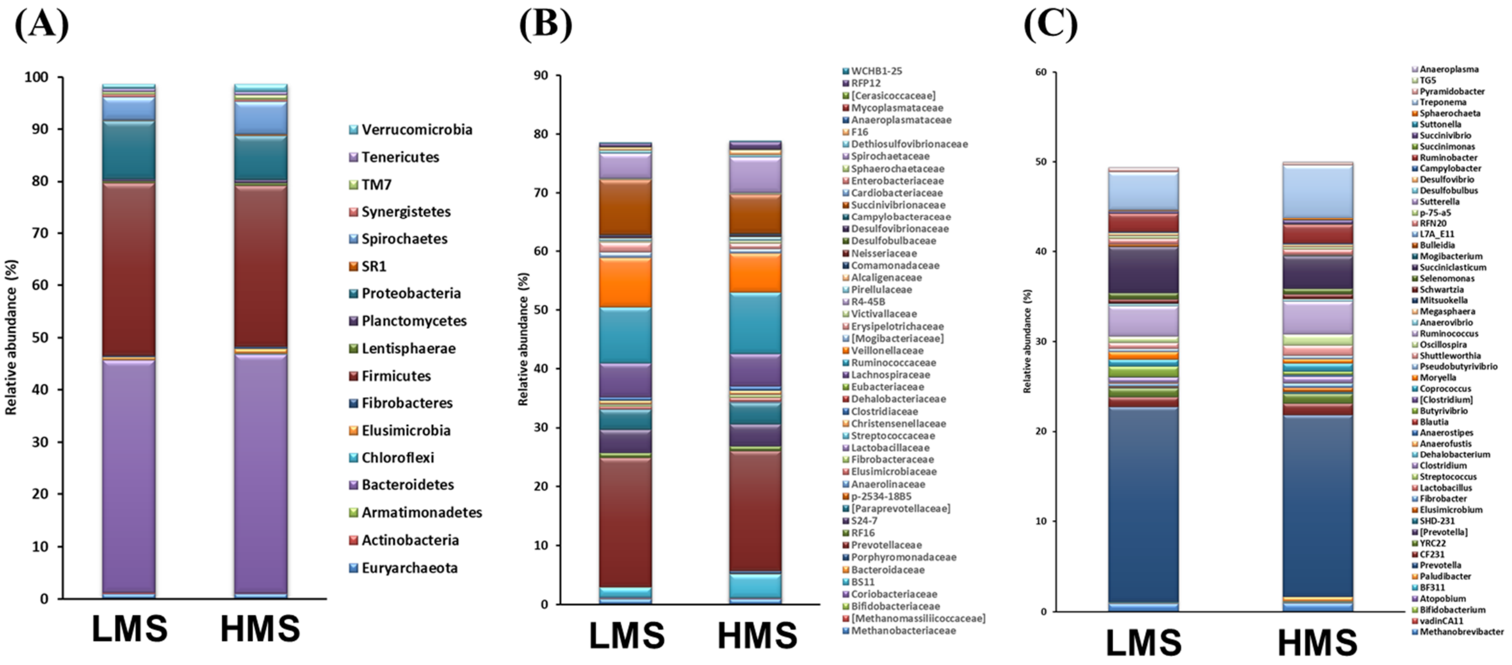

Figure S1. Relative abundance barplots of major classified (A) phyla, (B) families, and (C) genera (each taxon found in more than 50% of the 14 rumen samples) of prokaryotes detected in the rumen of Korean beef cattle.

© 2020 by the authors. Submitted for possible open access publication under the terms and conditions of the Creative Commons Attribution (CC BY) license (http://creativecommons.org/licenses/by/4.0/).
